# Arthrectomy of the Knee-Joint

**Published:** 1891-08

**Authors:** Herman Mynter

**Affiliations:** Professor of Operative and Clinical Surgery in Niagara University Medical College; 195 Franklin Street


### Buffalo Medical and Surgical Journal

Vol. XXXI. AUGUST, 1891. No. 1.

### Original Communications.

### ARTHRECTOMY OF THE KNEE-JOINT.[1]

By HERMAN MYNTER, M. D.,

Professor of Operative and Clinical Surgery in Niagara University Medical College.

A short *résumé* of the pathology of tuberculous affections of the knee-joint seems indispensable before discussing arthrectomy, and particularly the importance of the frequency of a local tuberculous focus in the epiphysis. If it should be proved that the majority of these chronic tuberculous joint affections originate from a local focus, there will be no excuse for overlooking and not removing it before the joint has become invaded. The trouble has been that a chronic tuberculous arthritis, whether originating in the bone or in the synovialis, gives, in the later stages, which formerly alone were operated, exactly the same pathological condition. If it starts as a local focus, and perforates into a healthy or partly obliterated joint, a tuberculous arthritis results. The synovialis becomes gradually changed into a tuberculous granulation tissue, the ligaments become softened, the cartilages ulcerated by pressure of the granulations (Volkman's ulcerative decubitus), while, at the same time, the Haversian canals become dilated, and filled with the same granulation tissue, by pressure of which the rest of the cartilages may become shed. And we have then total destruction of the joint, with caries of the epiphysis.

If, on the other hand, it originates as a synovitis, the result is exactly similar,—softening of the ligaments, ulceration of the cartilages, dilatation of the Haversian canals, with shedding of cartilages and caries of the epiphysis. That a carious epiphysis, from whatever cause, may offer a different picture at different points, is well known. Here we may have simply congestion, with

1. Read before the Medical Association of Central and Western New York, in Buffalo, June 2, 1891.

beginning dilatation; there the bone tissue has totally disappeared, and its place been taken by a granulation tissue; in other places we may find necrosis of the granulations from pressure, with formation of little irregular cavities, filled with cheesy pus and bone-spicula; in other places again, larger cavities with sequestra, and whether such a cavity is the original local focus or simply the result of the intense osteitis, it is impossible to decide. We have only one way of finding out, and that is in early operation, and my belief is, that in early operations we will, in almost every case, find a local bone-focus as the cause of the tuberculous arthritis, the prompt removal of which would have prevented the invasion of the joint.

I am aware that, so far, most surgeons disagree on this point.

Watson Cheyne states that, next to the vertebra, the knee-joint is most frequently the seat of tuberculous affections, and that there is a larger proportion of primary synovial disease than in the hip.

Willemin gives the following statistic: Below ten years of age, thirty-nine per cent. were of synovial origin, sixty-one per cent. of osseous origin; from ten to twenty years, forty-nine per cent. synovial, fifty-one per cent. osseous; above twenty years of age, thirty-three per cent. synovial, sixty-five per cent. osseous.

According to this statistic, the osseous origin becomes more prevalent with increasing age, and, consequently, the affections more obstinate. Sequestra were present in nineteen per cent. below ten years of age, in forty-nine per cent. above twenty years of age.

Koenig states that primary osseous and primary synovial diseases are about equally frequent in youth, but in old people osseous three times as frequent.

I can, of course, give no statistic to compare with those of these men, but will simply state, that in every case I have operated I have found a primary osseous lesion. In contrast to the hip, where the local focus most frequently occurs in the neck, the diaphysis, or acetabulum, the epiphysis of the femur and tibia are most frequently the seat of the local focus. Watson Cheyne never found, either in femur or tibia, a deposit beyond the epiphyseal line. The local focus occurs generally at the most exposed parts, and in the neighborhood of points of attachment of tendons and ligaments. The favorite seat is in the lower end of the femur, particularly the internal condyle, thereafter in the head of the tibia, and then in the patella. Small deposits or local foci are sometimes found immediately beneath the articular cartilages, through which they perforate. The color of the cartilage may indicate the place of the local focus, it being darker and somewhat spotted above it. If the foci are more deeply situated, they may either perforate into the joint or outwards outside the capsule, producing extra-articular abscesses. So much about the pathology. According to the different authors, at least one-half or more of the cases are of osseous origin. I believe that by far the greatest majority commence in this way. I have seen quite a number of early arthrectomies in Europe, and performed the operation a number of times myself, and I have yet to see the first case of synovial origin in childhood. In every case I have seen, a distinct local focus was found.

How, then, ought this disease to be treated ?

The ideal operation is, and would be, to remove the local focus in all cases in which the symptoms point to the formation of such I have lately had the opportunity of seeing two such cases, and will mention them here, as they illustrate the brilliant results of prompt operation and the dangers of delay.

The first patient, George Warner, ten years of age, and of healthy parentage, had a slight injury to his right knee during a visit to St. Louis, in January of this year. I was called to see him ten days later, found him limping slightly with the right foot, looking somewhat pale and peevish, and complaining of starting pains at night. By the objective examination, my first impression was that the knee was healthy, and I looked for symptoms of hip-disease. Finding none, I again carefully examined the knee, noted slight contraction of the hamstring tendons and some atrophy of the calf. Motion was free in the middle-ranges, but somewhat diminished in extreme flexion and extension. The measurement over patella gave one-half inch more than on left knee. On the middle of the lateral aspect of the internal condyle of the femur was found a spot as large as a nickel, on which tapping with the finger produced a most intense, deep-seated pain. The surface thermometer showed a temperature of 101° over this spot. Bodily temperature, 100°; pulse, 96.

I operated on him next day with the assistance of my friends, Drs Parmenter and Smith, trephined the internal condyle, and found the outer part of the bone hyperemic, and, in the center of the internal condyle, one-half inch from the surface, a small cavity as large as a cherry, filled with pus and tuberculous granulations, but no sequestrum. The cavity was scraped out with a sharp spoon, disinfected with coros. sublim., and plugged with iodoform gauze under an antiseptic dressing. The cavity healed by granulation in about three weeks, and perfect *restitutio ad integrum* followed. Have any of you any doubt that the starting pains at night, and the contraction of the hamstring muscles, indicated that the abscess was near the articular cartilages, and ready to perforate into the joint, and that I, by prompt operation, saved this child from a chronic tuberculous arthritis, with all its possibilities, such as arthrectomy, resection, etc. ?

The patient is present here, and you can satisfy yourselves about the perfect recovery.

The second case, Harold Kurtzman, was that of a little boy, two years of age, whom I saw on February 10, 1891. He had then limped, without known cause, with the left limb for about two weeks. The examination gave about similar results; some atrophy, slight enlargement over the head of the tibia, while over the internal condyle there was a tender spot, with increased surface temperature, starting pains at night, free motion in the middle-ranges, but slightly diminished extreme extension and flexion. I insisted upon operation, but could not persuade the parents, who consulted two other local surgeons who made light of the matter and merry over my diagnosis, and the patient passed out of my hands into theirs. Dr. Parmenter, who saw the patient with me, agreed with me in regard to diagnosis and treatment. I was called again May 13th, three months later, and learned that, meanwhile, two incisions had been made, during the last of which considerable pus (estimated at four tablespoons) and bone-tissue was removed. In order to make an exact diagnosis, I gave the child chloroform, again in the presence of my friends, Drs. Parmenter and Smith, and found a similar cavity in the head of the tibia as large as, or larger than, a hazelnut, and extending up close to the articular cartilage. I enlarged the opening, scraped the cavity out, and dressed it in a similar way. The child is rapidly recovering.

Have any of you any doubt that this operation might better have been performed three months earlier, and that the delay simply jeopardized the child's health and the functions of its limb? If we do not get hold of our patients early enough, and more or less destruction of the joint has taken place, we have the choice between resection and arthrectomy, or both. Formerly, the diseased bone ends were simply removed, the synovialis left intact. The result was anything but favorable. Long lasting suppurations were apt to follow, considerable shortening resulted, which increased during age, on account of the removal of the epiphyseal cartilage. Occasionally no osseous union occurred, and strong flexion was apt to occur from contraction of the flexor-muscles, necessitating, later on, osteotomies or amputation.

Of forty-five resections performed in Copenhagen between 1870 and 1880 (in the antiseptic period), only one had his wound healed by first intention, twelve had some suppuration, thirty-two copious suppuration and abscesses, thirty-one recovered, twelve died, and two were secondarily amputated ; but of the thirty-one recovered only one-fifth were without fistulas, and only eighteen gained a fair and useful limb.

A new step forward was occasioned by Volkman and Koenig's statement, that the disease frequently commenced as a local focus in the epiphysis, which, by perforating into the joint, produced the diffuse tuberculous inflammation, unless the joint had become more or less obliterated before the perforation. The former operation—resection—was, therefore, abandoned, and it was acknowledged necessary not only to find and remove the local focus, but the diseased synovial capsule just as well, which formerly had been left, and produced relapses, suppurations, etc. Otherwise the bones were left intact, and the lateral ligaments saved, if possible. This new operation was called arthrectomy, or erasion. But, to succeed well, early operation is necessary, in order not to meet too great destruction, and to avoid universal or local tuberculosis somewhere else. Yet, even after arthrectomy, the results are not always favorable, and a reaction has set in in favor of resection.

It was considered one of the advantages of this new operation that motion occasionally might be preserved, particularly in cases in which the cartilages and lateral ligaments were left intact, and the local focus had been removed without interfering with the articular cartilages.

Some motion, even if limited, would be of advantage in almost every joint in the body, but, as Plum, of Copenhagen, states, it is more than questionable whether motion is desired in the knee-joint or not. Plum has always observed flexion and valgus position of the knee following arthrectomy, and that in spite of all precautions with splints, plaster-of-Paris, etc., he thinks we are able, by either operation, to remove everything diseased, but that resection alone is able to produce a firm, bony anchylosis, and, therefore, is to be preferred. I do not know but what he is right. I have had the same trouble with the relapsing flexion in pure arthrectomy, and my best results have been in cases in which I, besides the synovial membrane, removed a thin slice of the femur and tibia, and got bony union. I will show you such a case here, which I operated about two years ago. I found an enormous local focus in the internal condyle of the femur, and had to remove the whole condyle. The joint being totally destroyed, the synovial membrane was removed, and a thin slice of bone taken from the denuded tibia and external condyle of the femur. The consulting surgeons present advised, after having seen the condition, that I immediately amputate the leg. Nevertheless, it healed by a moist bloodclot in less than two months without suppuration, and, as you see, he has a good, firm and useful leg.

In another patient, also present here, and operated four months ago, I found a local focus in the internal condyle of the tibia, which had perforated into the joint. I made here a pure arthrectomy, and have had considerable trouble with preventing contraction. She is still obliged to use plaster-of-Paris on that account.

What shall be done with the patella?

If resection is performed, it is of no further use, and may just as well be removed. If arthrectomy pure and simple is done, it is of use, as the extensor quadriceps femoris will, in some measure, counterbalance the flexor muscles. If no local focus should be found, relapses may occur, but as the joint proper has been removed, it will be in the form of an osteitis, and not an arthritis, and the tuberculous granulations are prevented from spreading far and wide.

I do not know that I can finish this short paper in any better way than by adding the conclusions of Ollier, of Lyons, France, as published in the Transactions of the International Medical Congress, held in Copenhagen in 1885, and with most of which I agree:

1. Articular tuberculosis presents a number of clinical varieties which demand a different treatment. Some are curable by arthrectomy or local destruction of the tuberculous products without sacrificing the healthy bone-tissue, others demand the removal of the diseased epiphysis, others, again, amputation.

2. Local, or apparently local, tuberculosis may continue indefinitely as such, and without tending to become general.

3. Conservative operations are indicated in local and circumscribed tuberculosis. The operation to be chosen depends upon the duration and extension of the lesion, the point in question being not to leave behind a focus from which by-and-by a secondary infection may occur.

4. Spontaneous recovery of osseous and articular tuberculosis occasionally occurs in infancy and youth by elimination. By surgically removing the tuberculous products, we follow the way indicated by nature. In adults, spontaneous recovery is much more rare, and complications of internal organs much more frequent.

5. Conservative operations are particularly indicated in infancy and youth, and more especially those operations which do not remove the joint cartilage, in order not to interfere with the growth of the bone. Amputations are most often necessary in mature or older age.

6. The surgeon ought always to be guided by the idea of early removing what is necessary, except in tuberculous affections in adults. Here amputation is often preferable.

7. In spite of antiseptics, arthrectomy is a more serious and less efficacious operation than a typical resection, and there is always fear of a local relapse. It is more apt to be followed by general tuberculosis than amputation or resection, by which operations the local foci are removed.

8. The rule to remove all tuberculous products does not apply to simply chronic inflammatory products.

By removing all fibrous tissues around a joint, defective functional results are often obtained.

9. By amputation we prevent best secondary infection of the wound, although even this operation is not absolutely a radical operation, as the deeper and more distant lymphatic glands may have become infected previously. Amputation is particularly indicated in osteo-arthritis of the lower extremity.

10. After a successful resection, when a cicatricial tissue has taken the place of the fungous granulations, the chances of relapse are about the same as after amputation, but the patient has the advantage of having retained his limb.

195 Franklin Street.

---

Diarrhea.—Dispensary prescription:

℞—Salol ........................................ dr. ij.
Bismuthi subnitratis,......................... dr. iv.
Mist. cretæ, q. s. ad.........................f. oz. iij.

M. Sig.—One teaspoonful every two hours.

